# What Is Gluten—Why Is It Special?

**DOI:** 10.3389/fnut.2019.00101

**Published:** 2019-07-05

**Authors:** Peter Shewry

**Affiliations:** Rothamsted Research, Harpenden, Hertfordshire, United Kingdom

**Keywords:** wheat, gluten, coeliac disease, protein, prolamin, gliadin, gluten, ATI

## Abstract

Wheat gluten has an immense impact on human nutrition as it largely determines the processing properties of wheat flour, and in particular the ability to make leavened breads, other baked products, pasta and noodles. However, there has been increasing interest in wheat gluten over the past two decades because of its well-established role in triggering coeliac disease, and its perceived role in other adverse reactions to wheat. The literature on wheat gluten is vast and extends back over two centuries, with most studies focusing on the structures of gluten proteins and their role in determining the functional properties of wheat flour and dough. This article provides a concise account of wheat gluten, focusing on properties, and features which are relevant to its role in triggering coeliac disease and, to a lesser extent, other gluten-related disorders. It includes descriptions of the biological role of the gluten proteins, the structures and relationships of gluten protein families, and the presence of related types of protein which may also contribute to functional properties and impacts on health. It therefore provides an understanding of the gluten protein system at the level required by those focusing on its impact on human health.

## Introduction

Wheat gluten was one of the earliest proteins to be studied scientifically, by Jacopo Beccari (Professor of Chemistry at the University of Bologna) in his article “De Frumento” (Concerning Grain) in 1745 (1, 2). It has since been studied in great detail by cereal chemists, because of its role in underpinning the ability to make leavened bread, other baked goods, pasta, and noodles. These properties are only shared to a very limited extent by related cereals (barley and rye). Hence, gluten underpins the production of staple foods for a substantial proportion of the global population, particularly in temperate zones.

Although gluten was identified as the trigger for coeliac disease almost 70 years ago (3), interest in gluten outside the scientific community was limited to those unfortunate enough to suffer from coeliac disease until early in the present century, which has seen an explosion of interest, particularly in the popular press and social media. As an example, a “Google” search carried out in December 2018 gave almost 400 million hits in less than a minute. This interest relates, of course, to the proposed role of gluten in triggering a range of adverse reactions, with substantial proportions of the population in many countries choosing to adopt a gluten-free, or low-gluten, diet. However, despite this massive interest few people have a clear understanding of gluten itself: what is it, what is the origin, why is it special?

This article, which forms part of the Special Research Topic “Gluten, from Plant to Plate: Implications for People with Celiac Disease,” therefore, provides a broad account of wheat gluten including its synthesis and deposition in the developing grain, the structures, and evolutionary relationships of its component proteins, and its unique properties which are exploited in grain processing, focusing on features which are relevant to its role in triggering coeliac disease. It does not cover other impacts of wheat proteins on human health, notably allergy, and non-coeliac gluten sensitivity (NCGS) which are discussed in other recent review articles (4, 5).

## What Is Gluten?

### Gluten Is Defined Based on Its Origin and Solubility

Gluten is classically defined as the largely proteinaceous mass which remains when a dough made from wheat flour and water is gently washed in an excess of water or dilute salt solution to remove most of the starch and soluble material (6). The remaining material, which has been described as “rubbery,” comprises about 75–80% protein on a dry matter basis, depending on how well the material is washed. Hence “gluten proteins” are defined as those present in this mass and, because similar material cannot be isolated from doughs made with flours from other cereals, gluten proteins are restricted to the grain of wheat (species of the genus *Triticum*). However, related proteins are present in other cereals (as discussed below) and these are frequently referred to as gluten in the non-specialist literature and the wider popular media.

More correctly, gluten and related proteins from other cereals are classified as “prolamins.” This name was coined by T.B. Osborne, the father of plant protein chemistry who worked at the Connecticut Agricultural experiment station from 1886 till 1928. During this period he published some 250 papers, including studies of seed proteins from 32 species. This allowed him to develop a broad classification of proteins based on their extraction in a series of solvents (7). This extraction is often performed sequentially (and called “Osborne fractionation”) with the four Osborne fractions being called albumins (soluble in water), globulins (soluble in dilute saline), prolamins (soluble in 60–70% alcohol), and glutelins (insoluble in the other solvents but may be extracted in alkali). The first two fractions are readily distinguished and the names are still in use, while prolamins were recognized as a defined group present only in cereal grains with the name being based on their high contents of proline and amide nitrogen (now known to be derived from glutamine). This fraction is given specific names in different cereal species: gliadin in wheat, hordein in barley, secalin in rye, zein in maize etc.

However, the final fraction (glutelin) is more difficult to define, as it effectively comprises all proteins which are insoluble in the three previous solvents but can be solubilized under conditions of extreme pH. In fact, glutelins are now known to comprise a mixture of unrelated proteins, including insoluble structural and metabolic proteins such as those bound to membranes and cell walls. However, these proteins are only present in small amounts and in wheat (and most other cereals) the major glutelin components are in fact prolamin subunits which are not extractable with alcohol/water mixtures due their presence as high molecular mass polymers stabilized by inter-chain disulphide bonds. In wheat these proteins are called glutenin and are present in about equal amounts to the alcohol-soluble gliadins, the two groups comprising gluten.

### Gluten Proteins Are the Major Storage Protein Fraction

**Figure 1:**
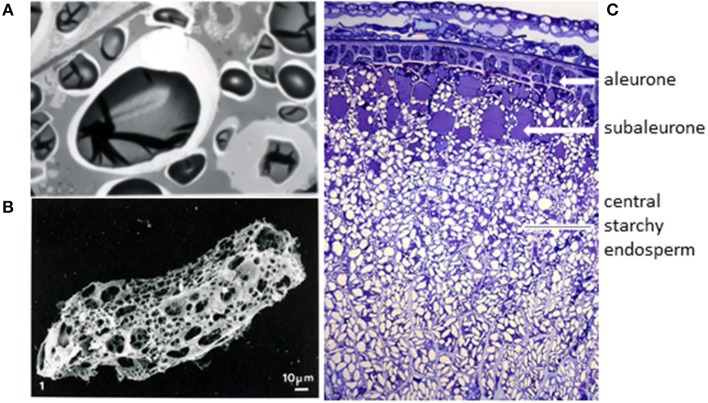
The origin of wheat gluten. **(A)** Transmission electron microscopy of starchy endosperm cells at a late stage of grain development (46 days after anthesis) shows that the individual protein bodies have fused to form a continuous proteinaceous matrix. Taken from Shewry et al. (9) with permission, provided by Dr. M. Parker (IFR, Norwich, UK). **(B)** Digestion of a flour particle to remove starch reveals a continuous proteinaceous network. Taken from Amend and Beauvais (10) with permission. **(C)** Transverse section of the lobe region of a developing wheat grain stained with Toluidine Blue to show the tissue structure and deposited protein (in blue). Figure kindly provided by Cristina Sanchis Gritsch and Paola Tosi (Rothamsted Research).

Gluten proteins are the major group of proteins which are stored in the grain to support germination and seedling development. They are restricted in distribution to the starchy endosperm cells of the grain, and have not been detected in any other tissues of the grain or plant. Their pathway and mechanisms of synthesis and deposition have been studied in detail [see Tosi (8)] but two points are particularly relevant here. Firstly, they are initially deposited in discrete protein bodies, which fuse during the later stages of grain development to form a continuous matrix surrounding the starch granules (Figure 1A). This matrix forms a continuous protein network within the cell, which can be revealed when the starch is removed from a flour particle by enzyme digestion (Figure 1B). It is easy to envisage how the protein networks present in the individual cells can be brought together during dough mixing to form the continuous gluten network in dough.

The second important point is that gluten proteins are not uniformly distributed in the starchy endosperm cells, but enriched in the outer 2 to 3 layers of cells (which are called the sub-aleurone cells). This is illustrated in Figure 1C, which shows a section of the starchy endosperm cells and outer layers from the lobe of the grain at a late stage of development stained with toluidine blue to show protein. In fact, Kent (11) calculated that the protein content of the cells of the starchy endosperm varies by over 4-fold, from 45% in the sub-aleurone cells to 8% in the central region. Furthermore, the gluten protein composition also varies, with the percentage of high molecular weight glutenin subunits (HMW subunits) increasing and the proportion of low molecular weight (LMW) subunits and gliadins (except for ω-gliadins) decreasing (these protein types are discussed below) (12). These gradients in composition are reflected to some extent in the contents and compositions of gluten proteins in the flour streams produced by commercial roller milling, meaning that these fractions may also vary in their impact on health (13).

#### Implications for Coeliac Disease

Fractionation by conventional milling combined with pearling (abrasion) or peeling (friction) could lead to flour streams that are enriched or depleted in coeliac-active proteins. The use of vital gluten (which is produced commercially for fortification of food products) also has implications. This will contain all of the gluten proteins present in the flour of origin, but may also contain other biologically active proteins as “co-passengers.”

## Gluten Proteins

### Gluten Comprises Several Related Families of Proteins Encoded by Multigene Families

The gluten protein fraction comprises a complex mixture of components which can be separated into groups by electrophoresis. Electrophoresis of the gliadins at low pH separates four groups of bands, called (in terms of decreasing mobility) α-gliadins, β-gliadins, γ-gliadins, and ω-gliadins. However, comparisons of amino acid sequences show that the α- and β-gliadins form a single group, sometimes called α-type gliadins.

The glutenin polymers are too big to be separated by conventional electrophoresis, but reduction of the inter-chain disulphide bonds that stabilize the polymers allows the subunits to be separated by sodium dodecylsulphate polyacrylamide gel electrophoresis (SDS-PAGE) into two groups of bands, called the HMW and LMW subunits. The latter group can be further sub-divided into a major group of components (B-type LMW subunits) and two minor groups (C-type and D-type).

Comparisons of amino acid sequences of these groups of gluten protein components clarifies their relationships, showing that the HMW subunits and ω-gliadins form discrete groups, with the α-gliadins, γ-gliadins, and B-type LMW subunits forming a third group. The minor groups of C-type and D-type LMW subunits appear to be modified forms of gliadins in which mutations to form cysteine residues allow their incorporation into glutenin polymers, with the C-type LMW subunits being modified α-gliadins or γ-gliadins and the D-type modified ω-gliadins. This classification is summarized in Table 1, which also shows their relative amounts and summarizes their characteristics (molecular masses and partial amino acid compositions).

**Table 1:** Summary of the types and characteristics of wheat gluten proteins [based on Shewry and Halford (14)].

**Gluten protein type**	**Molecular mass**	**% total gluten fraction**	**Polymers or monomers?**	**Partial amino acid composition (mol %)**
**HMW prolamins**				
HMW subunits	65–90,000	6–10	Polymers	30–35% glutamine, 10–16% proline, 15–20% glycine, 0.5–1.5% cysteine, 0.7–1.4% lysine
**S-rich prolamins**				
α-gliadins	30–45,000	70–80	Monomers	30–40% glutamine, 15–20% proline, 2–3% cysteine, <1% lysine
γ-gliadins				
B-type and C-type LMW subunits			Polymers	
**S-poor prolamins**				
ω-gliadins	30–75,000	10–20	Monomers	40–50% glutamine, 20–30% proline, 0–0.5% phenyl alanine, 0–0.5% lysine, 0 cysteine, 1 cysteine residue in D-type LMW subunits
D-type LMW subunits			Polymers	

Table 1 also groups the types of gluten proteins discussed above into three “families” (the HMW, sulfur(S)-rich, and S-poor prolamins), which were defined about 30 years ago based on emerging sequence data (15). This classification remains valid despite the vast increase in our knowledge of gluten protein sequences over the past few decades. For example, in May 2015 Bromilow et al. (16) retrieved over 24,000 sequences related to gluten proteins from the UniProt database. Removal of redundant, partial and mis-assigned sequences allowed the assembly of a curated database of 630 sequences.

The retrieval of over 600 sequences of gluten proteins does not, of course, mean that individual wheat genotypes contain this number of gluten proteins. Although the precise number of gluten proteins present in mature seed has not been determined, examination of two-dimensional (2D) electrophoretic separations indicates that the number of gluten proteins present in detectable amounts is probably between 50 and 100. This is consistent with the recent study of Bromilow et al. (17), who identified 63 gluten proteins in a single cultivar, using mass spectrometry and a curated sequence database (16). However, this study identified eight individual HMW subunit proteins, which is twice the number known to be present in the cultivar studied. This highlights the problems inherent in identifying gluten proteins based on short peptide sequences.

Although the prolamin groups discussed above undoubtedly account for the vast majority of the gluten proteins, recent work has shown that small amounts of a further type of gluten protein are present. These have been defined as δ-gliadins, although sequence comparisons indicate that they form part of the wider family of γ-prolamins (being closest in sequence to the γ3-hordeins of barley) (18, 19). Proteomic analysis indicates that they account for 1.2% of the total normalized spot volume in grain of Chinese Spring wheat (20).

### Molecular Basis for Gluten Protein Polymorphism

The large numbers of individual gluten proteins present in single genotypes, and the 10-fold greater number of sequences in databases, arises from three factors: the presence of multigene families, the high level of polymorphism between genotypes and, to a more limited extent, post-translational modification. It is therefore, necessary to consider these factors in turn.

Common wheat (*Triticum aestivum*), which includes modern bread wheat and spelt, is a hexaploid species, with three genomes (called A, B, and D) derived from related wild grasses. Only two of these genomes (A and B) are present in the tetraploid durum (pasta) wheat and emmer (forms of *Triticum turgidum*) while einkorn (*Triticum monococcum*) is diploid with only the A genome. Gluten proteins are encoded by loci on the group 1 and group 6 chromosomes of all three genomes, meaning that the gluten fraction can be expected to comprise more individual protein components in common wheat than in the other species. A detailed discussion of the genetics of gluten proteins is outside the scope of this article, but the reader can refer to Shewry et al. (21) for a detailed account.

Furthermore, all of the gluten protein loci comprise multiple genes. The simplest loci are the *Glu-1* loci which are located on the long arms of the group 1 chromosomes. Each of these loci comprises two genes which encode two types of HMW subunit of glutenin (called x-type and y-type). However, because not all of the *Glu-1* genes are expressed in all genotypes, the number of HMW subunit proteins in cultivars of bread wheat varies from 3 to 5 (22). Because of the simple genetic system, and the fact that the HMW subunits have been studied in more detail than most groups of gluten proteins, it is possible to define alleles at all three loci. Thus, the widely occurring pairs of subunits called 1Dx2 + 1Dy12 and 1Dx5 + 1Dy10 are alleles, while the pairs of subunits called 1Dx2 + 1Dy12 and 1Bx7 + 1By9 are homeoalleles (alleles on different genomes). The greater complexity of other gluten protein loci makes it much more difficult to recognize allelic forms of genes and proteins, although detailed analyses of allelic variation in LMW subunits have been reported [reviewed by Juhász et al. (23)].

However, whereas the individual HMW subunits can be assigned to sequenced genes, this is very difficult, if not impossible, for many other gluten proteins because of the complexity of the loci. For example, Huo et al. (19) assembled sequences of the α-gliadin loci on the three genomes of bread wheat, showing a total of 47 genes of which 26 encoded intact full-length protein products. Similarly, Qi et al. (24) reported the sequences of 29 putatively functional γ-gliadin genes (encoded by genes at the *Gli-1 loci* on the short arms of the group 1 chromosomes) in a single cultivar. Further information on the structures of the gluten protein multigenic loci are being provided by genome analysis [see, for example, (5, 25, 26)].

It is also likely that the numbers of expressed genes vary between genotypes. Thus, the high polymorphism in gluten protein composition observed between genotypes may arise both from variation in the numbers of expressed genes, and variation in the sequences of the encoded proteins.

A third factor which may contribute to protein polymorphism is post-translational modification. Gluten proteins contain between about 20 and 50 mol % of glutamine residues so post-translational deamidation has long been recognized as a possibility. It may, for example, account for the fact that HMW subunits often form “trains” of spots in 2D electrophoresis, while Dupont et al. (27) reported the presence of HMW subunit sequences in 43 spots separated on 2D gels. However, the extent of deamidation has never been quantified. Other proposed modifications, such as glycosylation (28) and phosphorylation (29) have not been substantiated by further studies. Other types of post-translational modification may include cyclisation of N-terminal glutamine to give pyroglutamate (which is likely to be responsible for many gluten proteins having “blocked” N-termini), differential processing of the signal peptide (30) and proteolysis by legumain-like asparaginyl endoproteinase (31).

Finally, the proportions of gluten proteins may also be affected by the environment, including temperature during grain development and availability of nutrients (nitrogen and sulfur) [reviewed by DuPont and Altenbach (32) and Altenbach (33)]. In particular, increases in the proportions of gliadins occur under high nitrogen availability and of ω-gliadins when nitrogen availability is high but sulfur is limiting.

#### Implications for Coeliac Disease

Protein polymorphism is clearly a challenge for attempts to eliminate “toxic” proteins and to develop coeliac-safe wheats, whether by exploiting natural variation or by genetic engineering/genome editing.

Effects of environment on gluten protein composition will also have impacts on the abundances of specific coeliac disease epitopes.

### Gluten Proteins Contain Unique Repetitive Domains

The most important characteristic of wheat gluten proteins in relation to their role in coeliac disease is the presence of protein domains comprising repetitive sequences. The domains vary in extent, but generally account for between about 30 and 50% of the protein sequence in S-rich gliadins and LMW subunits, between 75 and 85% in HMW subunits, and almost the whole protein in ω-gliadins [reviewed by Shewry et al. (34)]. They comprise tandem repeats of short peptides comprising between three and nine amino acid residues, and may be based on tandem repeats of one motif or tandem and interspersed repeats of two or more motifs.

The most widely studied repetitive sequences are those present in the HMW subunits of glutenin. These comprise repeats based on three motifs: the hexapeptide PGQGQQ, the nonapeptide GYYPTSPQQ or GYYPTSLQQ, and in x-type subunits only, a tripeptide GQQ (P, proline; G, glycine; Q, glutamine, Y, tyrosine; P, proline; T, threonine, S, serine; L, leucine) (34). The motifs present in the other groups of gluten proteins are generally less well-conserved and the identification of consensus motifs is more subjective than in the HMW subunits, but all are rich in proline and glutamine, for example, PQQPFPQQ (F, phenyl alanine) in γ-gliadins. It should be noted that these sequences are responsible for the characteristic amino acid compositions of the whole proteins, notably the high contents of glutamine (35–55 mol%) and proline (10–25 mol%) in all groups of prolamins, high glycine in HMW subunits (11–12 mol%), and high phenyl alanine (about 11 mol%) in ω-gliadins [reviewed by Shewry et al. (34)].

The repeated sequences may also be responsible for the unusual solubility properties of gluten proteins. Although glutamine is a hydrophilic amino acid, the regularly repeated glutamine residues in gluten proteins are considered to form protein:protein hydrogen bonds resulting in insolubility in water (as discussed by Belton (35) for HMW subunits). However, in most gluten proteins, all of the cysteine residues, which may form interchain or intrachain disulphide bonds, are located in the non-repetitive domains.

**Figure 2:**
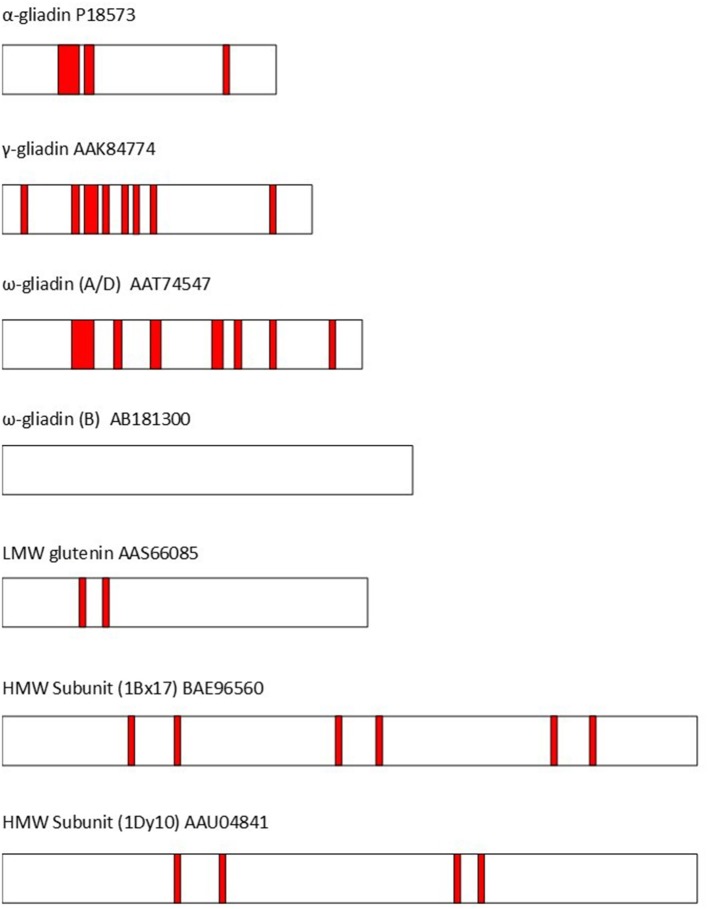
The distribution of T-cell epitopes (shown as red bars) in representative wheat gluten proteins (identified by GenBank accession codes). The epitopes are based on Sollid et al. (36). **α**-gliadin P18573: DQ2.5-glia-α1a, DQ2.5-glia-α1b, DQ2.5-glia-α2, & DQ8-glia-α1. γ-gliadin AAK84774: DQ2.5-glia-ω1/hor-1/sec-1, DQ8-glia-γ1a, DQ8-glia-γ2, DQ8-glia-γ4c, & DQ8-glia-γ5. ω-gliadin (A/D) AAT74547: DQ2.5-glia-γ5, DQ8-glia-γ1a, DQ2.5-glia-ω1/hor-1/sec-1, DQ8-glia- γ1b, & DQ2.5-glia- γ3. ω-gliadin (B) AB181300 no coeliac toxic epitopes present. LMW subunit AAS66085:DQ2.5-glut-L1. HMW Subunit (1Bx17) BAE96560: DQ8.5-glut-H1. HMW Subunit (1Dy10) AAU04841: DQ8.5-glut-H1. Modified from Shewry and Tatham (37).

The repetitive sequences also play a crucial role in triggering coeliac disease. In fact, all of the 31 “coeliac disease relevant T-cell epitopes” listed by Sollid et al. (36) are present in the repetitive domains of wheat or related cereals (barley, oats, rye) and all groups of gluten proteins (gliadins and glutenins) contain epitopes. Nevertheless, some individual proteins within these groups may lack recognized coeliac epitopes (although the current list of epitopes is considered to be incomplete). This is illustrated by Figure 2 (37) and discussed in detail by Shewry and Tatham (37), Gilissen et al. (38), and Juhasz et al. (5).

#### Implications for Coeliac Disease

As discussed above, all of the coeliac-toxic epitopes in wheat gluten proteins are present in the repeated sequences, with multiple epitopes present in some repetitive domains. This clearly poses a significant challenge for attempts to “remove” epitopes by transgenesis or gene editing.

## The Prolamin Superfamily

The prolamins, including wheat gluten proteins, were historically defined as a unique class of proteins restricted to the grain of cereals and related grass species, based on their unusual amino acid compositions and solubility properties (7) and this dogma was not questioned until the increasing availability of protein sequence data allowed wider comparisons to be made. The first report that prolamins were related to a wider range of proteins was in 1985, when Kreis et al. (39) showed the sequences present in the cysteine-rich non-repetitive regions of prolamins were related to sequences in two other groups of seed proteins: cereal inhibitors of α-amylase and trypsin (now called ATIs) and 2S albumin storage proteins of dicotyledonous seeds. Although these groups of proteins have little sequence identity with each other or with prolamins, the homology was based on very high conservation in the numbers and spacing of cysteine residues. Further comparisons exploiting the vast increase in sequence data have since identified several other groups of related proteins, which are together referred to as the “prolamin superfamily.”

The prolamin superfamily includes proteins which are not restricted to cereals and grasses, and present in tissues other than seeds (40). However, several types are present in wheat grain, and may contribute to the functional properties and role in diet and health (34). They are therefore, briefly discussed here and summarized in Table 2.

**Table 2:** Wheat grain proteins of the prolamin superfamily (based on literature discussed in the text).

**Protein group**	**Molecular mass**	**Characteristics**	**Abundance**	**Functional properties/impact on health**
Farinins	17,000–30,000	Correspond to avenin-like proteins and LMW gliadins	Not determined	Transgenic expression results in improved mixing properties
Purinins (low molecular weight gliadins)	17,000–19,000	Possibly correspond to “ancestral” type of prolamin	Not determined	Behave like gliadins in dough
Puroindolines a and b	13,000	Tryptophan-rich loop region which may be involved in binding to starch granule surface	0.029–0.060 % dry wt of Pin a and 0.004–0.031 % dry wt. of Pin b in wholemeal flour	Determine about 75% of the variation in softness in European wheats
Grain softness protein (GSP)	~15,000	Associated with the starch granule surface	Not determined	Small effect on grain softness
+				
Arabinogalactan peptide (AGP)	23,000	15 residue peptide *o*-glycosylated with arabinogalactan chains at 3 hydroxyproline residues	0.39% dry wt. white flour	Prebiotic properties *in vitro*
Non-specific lipid-transfer proteins (LTP)	9,000 (LTP1) + 7,000 (LTP2)	Bind and transport lipids *in vitro* Concentrated in aleurone layer and embryo	Not determined	LTP1 is a food and respiratory allergen
α-amylase/trypsin inhibitors (ATIs)	12,000 to 16,000	Monomeric, dimeric, and tetrameric forms, some subunits inhibit trypsin or α-amylase	0.34–0.41% dry wt. of wholemeal flour	Include respiratory and food allergens, putative links to coeliac disease, NCWS, and other adverse reactions to wheat Contribute to pasta-making quality

### Farinins and Purinins

It has been known for many years that wheat flour contains proteins with molecular masses below 30 kDa which are related to gluten proteins, including types described as globulins, LMW gliadins, and avenin-like proteins. Kasarda et al. (41) have recently discussed the relationships of these proteins and suggested that they should be classified into two types, which they termed farinins and purinins. Both are more closely related to gliadins than the other protein types discussed below, but lack the repeated sequences which are typical of gliadins. Hence they have been classed as globulins based on solubility. The farinins correspond to the avenin-like proteins (defined based on homology with the avenin proteins of oats) with two types called a (which correspond to LMW gliadins) and b (42). These groups differ in that the b-type proteins contain a duplicated sequence of about 120 residues, resulting in a higher molecular weight (about 30 kDa compared with 17 kDa). The b-type proteins are associated with the surface of the starch granule and are post-translationally cleaved to give two subunits (11 and 19 kDa) linked by a single disulphide bond (41). Ma et al. (43) showed that over-expression of a transgene encoding a b-type protein resulted in improved flour mixing properties and an increased proportion of large glutenin polymers, presumably due to their ability to form inter-chain disulphide bonds.

The LMW gliadins/purinins have masses of about 17–19 kDa (44) and are more closely related to the γ-gliadins in sequence (41, 45). They may, perhaps, be considered to be similar to the “ancestral” prolamin proteins, before they diverged due to the development and amplification of the repetitive sequence domains. Mixing of heterologously expressed proteins into dough showed similar effects to the incorporation of gliadins (45).

### Puroindolines (Pins) and Grain Softness Protein (GSP)

Hardness is one of the major characteristics used to divide wheat into end use classes. It is determined by the *Hardness* (*Ha*) locus on the short arm of chromosome 5D of bread wheat, although the name is misleading because the encoded genes actually determine softness. This locus is not present in durum wheat which is therefore ultrahard. The *Ha* locus comprises three genes (46), encoding proteins called puroindoline a (Pin a), puroindoline b (Pin b) and grain softness protein (GSP). The mature Pin a and Pin b proteins comprise about 120 amino acid residues including 10 cysteine residues which form inter-chain disulphide bonds. They also contain five (in Pin a) or three (in Pin b) tryptophan residues which are grouped together in the sequences. Comparison of wholemeal flours of 40 wheat cultivars (19 soft and 21 hard) grown on four French sites showed 0.029–0.060 % dry wt of Pin a and 0.004–0.031% dry wt of Pin b (47). Differences in the expression of these proteins, and/or their amino acid sequences, account for about 75% of the variation in grain hardness in bread wheat (48).

The third gene at the *Ha* locus encodes a protein which is cleaved post-translationally, probably in the vacuole by a similar legumain-type asparaginyl endoproteinase to the enzyme(s) responsible for proteolysis of gluten proteins (as discussed above). This releases a 15 residue peptide from the N-terminus (49). This peptide contains three proline residues which are hydroxylated to give hydroxyprolines and then *o*-glycosylated with arabinogalactan chains to give a mass of about 23 kDa (50). The resulting “arabinogalactan peptide” (AGP) accounts for about 0.39% of the dry weight of white flour (50) and is readily fermented by the colonic microflora (51). The remaining part of the protein, termed “grain softness protein” (GSP), may contribute to hardness to a limited extent [by about 10 units measured by the Perten Single Kernal Characterization System (SKCS)] (52), but the biological roles of AGP and GSP are not known.

### Non-specific Lipid Transfer Proteins (LTPs)

Unlike the other proteins discussed here, LTPs are not restricted to seed tissues, or to cereals and other grass species. Although they were initially defined on their ability to transfer phospholipids between liposomes and membranes *in vitro*, their true physiological role is unknown with one possible function being to contribute to defense to biotic stresses. They occur in two classes, with masses of about 9 kDa (LTP1) and 7 kDa (LTP2) and are concentrated in the aleurone layer and embryo of the wheat grain [reviewed by Marion et al. (53)]. Many LTPs have been identified as allergens, in seeds, fruit, and pollen (53), with LTP1 of wheat contributing to both food allergy and Bakers' asthma (respiratory allergy to wheat flour) (54, 55).

### α-Amylase/Trypsin Inhibitors

Wheat inhibitors of α-amylase and trypsin have been studied for over 40 years, resulting in an extensive and somewhat confusing literature. This results partly from the complexity of the fraction but also from use of different nomenclatures, based on relative electrophoretic mobilities (the major components being called 0.19, 0.28, and 0.53), solubility in chloroform:methanol (called CM1 to CM17) and subunit structure (monomeric, dimeric, and tetrameric forms occurring) (56). Dupont et al. (27) used mass spectrometry of proteins separated by 2D electrophoresis to identify two spots corresponding to forms of the putative monomeric trypsin inhibitor(s) CM1/3, two related to the monomeric amylase inhibitor WMAI, two related to the homodimeric amylase inhibitor WDAI1, and nine related to subunits of the heterotetrameric amylase inhibitor WTAI (1 × CM1, 2 × CM2, 2 × CM3, 2 × CM16, and 2 × CM17). More recently, Geisslitz et al. (57) have used targeted LC-MS to quantify the amounts of the major ATIs (WDAI/0.19 + 0.53; WMAI1/0.28, CM2, CM3, CM16, and CM17), showing that they together accounted for 3.4–4.1 mg/g in wholemeal flour of bread wheat.

Wheat ATIs are well-characterized as wheat allegens, particularly in Bakers' asthma but also on ingestion of food [reviewed by Salcedo et al. (58)]. In addition, they have been studied widely over the past few years because of putative roles in other adverse reactions to wheat consumption, including coeliac disease, and non-coeliac wheat/gluten sensitivity (as discussed in other contributions to this special section).

ATIs have also been reported to contribute to the cooking quality of pasta, where they were initially reported to be glutenin components (called durum sulfur-rich glutenin, DSG) (59–61).

#### Implications for Coeliac Disease

Wheat grain contains many other proteins including other families of protease and amylase inhibitors, thionins, ribosome-inactivating proteins, and putative defense-related proteins with unknown functions [reviewed by Shewry et al. (34)]. All of these may be present in food products, present either in flours or as “contaminants” in vital gluten. However, the proteins discussed above share some properties which may be particularly relevant. Firstly, most are small globular proteins which are tightly folded and stabilized by multiple interchain disulphide bonds. Hence, they are particularly stable to heating during food processing and to degradation in the gastro-intestinal tract: although proteolysis may occur, the proteins will not disintegrate because the fragments are held together by the disulphide bonds. Secondly, they may interact strongly with gluten proteins and hence be present in vital gluten. These interactions may be stabilized by non-covalent forces, such as the LMW gliadins/purinins, or by disulphide bonds formed either during grain development and maturation or re-arrangements during processing. Irrespective of the mechanism, the fact that they may be present in “gluten protein” fractions shows that they must be considered when interpreting studies carried out on human responses to wheat proteins.

## Gluten Proteins Have Unique Biophysical Properties Which Underpin Grain Processing

Several factors have contributed to the global success of wheat, one being its wide adaptability. However, the main reason why it is grown in preference to other cereal crops in many countries is the functional properties of wheat flour. As discussed above, wheat is the only cereal which can be baked to give leavened bread and other baked products, as well as pasta and noodles. The quality for these end uses is determined largely by the gluten proteins, which form a continuous network in dough. This network provides the cohesiveness required for making products such as pasta as well as the visco-elasticity required for breadmaking.

Despite a massive literature the molecular basis for the biophysical properties of gluten is still not completely understood, and it is not possible to provide a detailed discussion here. However, two points are particularly relevant. Firstly, the properties depend on the contributions of both the gliadins and glutenins, with the glutenin subunits forming large three dimensional networks stabilized by inter-chain disulphide bonds which interact with gliadins, and with other glutenin networks, by non-covalent forces, particularly hydrogen bonds. Secondly, the polymers are stabilized by a combination of forces. The importance of disulphide bonds is readily demonstrated as these can be disrupted using reducing agents, with catastrophic effects on functionality. The importance of hydrogen bonds is less easy to demonstrate, but Belton (35) has proposed that hydrogen bonds are particularly important in developing optimal protein interactions during dough mixing.

### Implications for Coeliac Disease

The clearest implication for coeliac disease is that any drastic modification to the composition of the gluten protein fraction and/or to the sequences of the individual subunits are likely to have effects on functionality. Although these effects are not easy to predict, that fact that bread making wheats have been selected for functional properties for almost a century suggests that most modifications will be detrimental. Thus, although it may be possible to produce “acceptable” loaves from modified lines of wheat in the laboratory and in small scale systems [see, for example, (62, 63)], this is a much greater challenge for large scale commercial production where profit margins are narrow and small differences in parameters such as loaf height, crumb texture, color and shelf life will affect the quality of the product and hence acceptability by consumers.

## Conclusion

Wheat gluten fulfills an essential biological role as the major grain storage protein fraction, and is the major determinant of the functional (processing) properties of the grain. It is a highly complex mixture of proteins, encoded by multigene families at multiple loci on the three genomes of bread wheat, with a high degree of polymorphism between genotypes. The individual proteins also have unusual structures, including extensive domains of repetitive sequences. In addition, a range of related proteins are present in the grain and may be present in isolated gluten fractions. All of these factors must be considered when studying the role of gluten in coeliac disease and other adverse responses to wheat consumption, and in designing strategies to develop safe types of wheat and wheat products.

## Author Contributions

PS wrote the whole paper. Part of Figure 1 was provided by colleagues.

### Conflict of Interest Statement

The author declares that the research was conducted in the absence of any commercial or financial relationships that could be construed as a potential conflict of interest.
